# Reply to Comment on Di Marco, N., Kaufman, J., Rodda, C.P. Shedding Light on Vitamin D Status and Its Complexities during Pregnancy, Infancy and Childhood: An Australian Perspective. *Int. J. Environ. Res. Public Health* 2019, *16* (4), 538, doi:10.3390/ijerph16040538

**DOI:** 10.3390/ijerph16091576

**Published:** 2019-05-06

**Authors:** Nelfio Di Marco, Jonathan Kaufman, Christine P. Rodda

**Affiliations:** 1 Women’s and Children’s Division, Sunshine Hospital, St Albans, VIC 3021, Australia; nelfiodm@gmail.com (N.D.M.); Jonathan.Kaufman@wh.org.au (J.K.); 2 Department of Paediatrics, University of Melbourne, Royal Children’s Hospital, Parkville, VIC 3052, Australia; 3 Australian Institute for Musculoskeletal Science, University of Melbourne, Sunshine Hospital, St Albans, VIC 3021, Australia

We thank the author(s) for their most informative letter in response to our article [1] published in IJERPH in February this year, highlighting yet another example of change in public health policy resulting in an increased incidence of rickets, from the United Kingdom. In the UK, Cod Liver Oil was no longer recommended for use in babies to prevent rickets, because of concerns about Vitamin A toxicity. The author(s) described the resulting resurgence in rickets following this public health recommendation. Both UK and Australian Public Health messages appear to have addressed, in the UK the risk of vitamin A toxicity with Cod Liver Oil, and in Australia promoting the benefits of “Breast is Best”, without due consideration of possible risks of implementation of such Public Health messages. The current Australian Guidelines based on Paxton and colleagues [2], recommend that exclusively breast fed infants with one other risk factor for vitamin D deficiency should receive vitamin D 400iu (10 µg) daily. Preparations containing Vitamin D alone rather than Cod Liver Oil have been promoted in Australia in recent years, for clarity and simplicity. There are currently numerous Cod Liver Oil preparations available in Australia on the internet, with very variable amounts of both vitamin A and Vitamin D. Although the Vitamin A amounts are safe, if taken as recommended, the vitamin D doses vary from 85 units (2.1 µg) to 450 units (11.25 µg) per daily dose. In comparison, Osteovit D [3] is available in several formulations containing Vitamin D alone and including 200iu (5 µg)/0.04 mL drop prophylaxis for infants and 2000 units (50 µg)/drop for treatment of vitamin D deficiency in older infants and children.
